# Long non-coding RNAs as promising biomarkers and therapeutic targets in cervical cancer

**DOI:** 10.1016/j.ncrna.2023.02.006

**Published:** 2023-02-21

**Authors:** Sema Begliarzade, Aferin Beilerli, Albert Sufianov, Rasim Tamrazov, Valentin Kudriashov, Tatiana Ilyasova, Yanchao Liang, Ozal Beylerli

**Affiliations:** a Republican Clinical Perinatal Center, Ufa, Republic of Bashkortostan, 450106, Russia; b Department of Obstetrics and Gynecology, Tyumen State Medical University, 54 Odesskaya Street, 625023, Tyumen, Russia; c Educational and Scientific Institute of Neurosurgery, Рeoples’ Friendship University of Russia (RUDN University), Moscow, Russia; d Department of Neurosurgery, Sechenov First Moscow State Medical University (Sechenov University), Moscow, Russia; e Department of Oncology, Radiology and Radiotherapy, Tyumen State Medical University, 54 Odesskaya Street, 625023, Tyumen, Russia; f Gastric Cancer Center, West China Hospital of Sichuan University, China; g Department of Internal Diseases, Bashkir State Medical University, Ufa, Republic of Bashkortostan, 450008, Russia; h Department of Neurosurgery, the First Affiliated Hospital of Harbin Medical University, Harbin, 150001, China

**Keywords:** Long non-coding RNAs, Cervical cancer, Abnormal regulation, Molecular regulation, Therapeutic target

## Abstract

Cervical cancer is the second most common cancer in women. The detection of oncopathologies in the early stages of development is a paramount task of modern medicine, which can be solved only by improving modern diagnostic methods. The use of screening for certain tumor markers could complement modern tests such as testing for oncogenic types of human papillomavirus (HPV), cytology, colposcopy with acetic acid and iodine solutions. Such highly informative biomarkers can be long noncoding RNAs (lncRNAs) that are highly specific compared to the mRNA profile and are involved in the regulation of gene expression. LncRNAs are a class of non-coding RNAs molecules that are typically over 200 nucleotides in length. LncRNAs may be involved in the regulation of all major cellular processes, including proliferation and differentiation, metabolism, signaling pathways, and apoptosis. LncRNAs molecules are highly stable due to their small size, which is also their undoubted advantage. The study of individual lncRNAs as regulators of the expression of genes involved in the mechanisms of oncogenesis cervical cancer can be not only of great diagnostic value, but, as a result, of therapeutic significance in cervical cancer patients. This review article will present the characteristics of lncRNAs that allow them to be used as accurate diagnostic and prognostic tools, as well as to consider them as effective therapeutic targets in cervical cancer.

## 1 Introduction

Cervical cancer is the second malignant tumor that seriously threatens women's health. There are more than 490,000 new cases of cervical cancer in the world every year. In recent years, the incidence of cervical cancer in my country has increased significantly and has a younger trend [1]. Cervical cancer is a disease in which multiple factors, multiple genes, and multiple links interact together to form a complex molecular regulatory mechanism. Surgical resection is the main treatment for early-stage cervical cancer, and the prognosis is good, while surgery combined with chemotherapy and radiotherapy is the main treatment for middle-advanced cervical cancer, and the prognosis is poor [2]. The diagnosis and treatment of cervical cancer lack specific indicators for monitoring tumor metastasis, judging prognosis, recurrence, and guiding individualized treatment. Therefore, finding ideal and effective tumor molecular markers is of great significance for improving the diagnosis and treatment of cervical cancer. At present, lncRNAs are popular tumor molecular markers in the field of life sciences. Chen Qiantao and other scholars used high-throughput lncRNAs chip technology to detect the changes of lncRNA expression profiles in cervical cancer tissue and normal cervical tissue, and detected a total of 30,586 lncRNAs. After cluster analysis and comparison, a total of 22,043 differentially expressed lncRNAs were found, of which 11,545 were up-regulated and 10,498 were down-regulated [3]. It shows that lncRNAs plays an important role in cervical cancer, and what function and how it plays a role in cervical cancer is worth exploring.

Most of the human genome can be transcribed, but less than 2% of the genome are protein-coding genes, and the rest of the genome is transcribed into non-coding RNA (non-coding RNA, ncRNAs). NcRNAs can be divided into two categories according to their molecular length, namely, short non-coding RNAs (small non-coding RNA, sncRNAs) with a length of less than 200 nt and long non-coding RNAs (long non-coding RNA, lncRNAs) with a length of more than 200 nt. According to the relative position of adjacent protein-coding transcripts, lncRNAs can be divided into sense lncRNAs, antisense lncRNAs, bidirectional lncRNAs, intragenic lncRNAs, and intergenic lncRNAs [4]. With the development of next-generation sequencing technology, the biological functions and behavioral mechanisms of lncRNAs in eukaryotes have been gradually elucidated. Studies have shown that lncRNAs can interact with DNA, RNA, and proteins, and regulate the biological processes of cells through different molecular mechanisms of DNA methylation, histone modification, and miRNA competitive inhibition [[5], [6], [7]]. LncRNAs are not only widely involved in the normal growth and development of the body, but also closely related to the occurrence and development of human diseases. Studies have shown that the expression of various lncRNAs changes significantly in the occurrence and development of cervical cancer and after treatment of cervical cancer [3,8]. Therefore, in-depth study of the relationship between lncRNAs and cervical cancer is expected to provide a new basis for the clinical diagnosis and effective treatment of cervical cancer.

## 2 Abnormal regulation of lncRNAs in cervical cancer

**Fig. 1:**
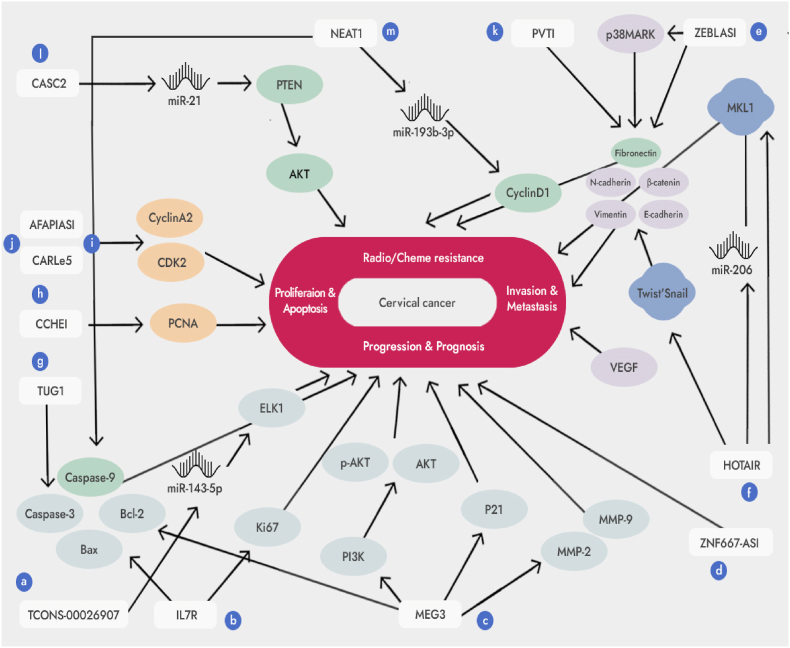
The role of long non-coding RNAs in cervical cancer. a: TCONS_00026907 regulates ELK1 to promote cervical cancer progression by inhibiting miR-143-5p. b: IL7R is an independent predictor of cervical cancer through the regulation of Bcl-2, caspase-3 and Ki-67; c: MEG3 inhibits cervical cancer progression by modulating PI3K/AKT/Bcl-2/Bax/P21 and PI3K/AKT/MMP-2/9 signaling pathways; d: ZNF667-AS1 is associated with overall survival, tumor size, and FIGO stage of cervical cancer and inhibits cell cloning and proliferation of cervical cancer; e: ZEB1-AS1 regulates EMT-associated E-cadherin to promote invasion and migration of cervical cancer cells through activation of the p38MAPK signaling pathway; f: HOTAIR promotes invasion and migration of cervical cancer cells by upregulating VEGF, MMP-9, EMT-associated proteins E-cadherin, β-catenin and vimentin, Snail and Twist transcription factors, and upregulating MKL1 through inhibition of miR206; g: TUG1 inhibits apoptosis of cervical cancer cells by regulating Bcl-2 and caspase-3; h: CCHE1 promotes cervical cancer cell proliferation through regulation of PCNA; i, j: AFAP1-AS1 and CARLo-5 promote cervical cancer cell proliferation by regulating cell cycle-associated CDK2 and CyclinA2 proteins; k: PVT1 contributes to cervical cancer resistance to chemotherapy through the regulation of EMT-related proteins E-cadherin, fibronectin and vimentin; l: Low expression of CASC2 regulated PTEN to increase cervical cancer resistance to chemotherapy by stimulating miR-21; m: NEAT1 regulates cyclin D1, caspase-3 and caspase-9 to increase cervical cancer resistance to radiotherapy by inhibiting miR-193b-3p.

LncRNAs play a role in many biological processes in cells. Studies have shown that various lncRNAs are expressed differently in cervical cancer and may be involved in growth, differentiation, migration, invasion, apoptosis, and other processes, thereby influencing the occurrence and development of cervical cancer [3,9]. The abnormal regulation of lncRNAs in cervical cancer is shown in Fig. 1.

### 2.1 LncRNAs affect the progression and prognosis of cervical cancer

Cervical cancer is a disease caused by the interaction of many factors, many genes and multiple connections through complex molecular regulatory mechanisms. More and more studies show that lncRNA can be used to diagnose and predict tumors [10,11]. Jean et al. found that TCONS_00026907 is abnormally expressed in cervical cancer using a lncRNA microarray. The expression of TCONS_00026907 is significantly upregulated in cervical cancer tissues, and this may promote the cell cycle process, proliferation, migration and invasion, as well as inhibit apoptosis. Mechanistic studies have shown that after TCONS_00026907 silencing, miR-143-5p expression was significantly increased and ELK1 target gene expression downstream of miR-143-5p was significantly reduced, thereby suppressing the development of cervical cancer [12] (Fig. 1a). In addition, from normal cervical tissue, cervical intraepithelial neoplasia (CIN) to cervical cancer tissue, Fan et al. [13] found that expression of inflammation-associated lncRNA receptor interleukin 7 (interleukin 7 receptor, IL7R) tends to increase. High IL7R expression is positively correlated with tumor size, FIGO stage, and lymph node metastasis, and patients with high expression have a shorter overall survival and poor prognosis. Cox regression analysis showed that IL7R can be used as an independent predictor of cervical cancer. Functional experiments show that interference with IL7R expression can inhibit cervical cancer growth In vitro experiments show that Bcl-2 expression is reduced and caspase-3 expression is increased, which can inhibit cervical cancer growth by promoting apoptosis. In vivo experiments also show that reduced expression of Ki-67 inhibits the growth of cervical cancer (Fig. 1b).

In contrast, the long-chain non-coding MEG3, ZNF667-AS1, was underexpressed in cervical cancer. Maternally expressed gene 3 (MEG3) is the first imprinted lncRNA discovered, which has the function of suppressing tumors, and it is located on chromosome 14q32. MEG3 is low expressed in cervical cancer and negatively correlated with FIGO stage, tumor size and lymph node metastasis [14]. Wang et al. showed by RT-PCR and Western blotting analysis that after overexpression of MEG3, the gene and protein expressions of PI3K, AKT, MMP-2, MMP-9 and Bcl-2 were all decreased, while the gene and protein expressions of Bax and P21 were all decreased raised [15]. This suggests that MEG3 inhibits cervical cancer progression by regulating PI3K/AKT/Bcl-2/Bax/P21 and PI3K/AKT/MMP-2/9 signaling pathways in cervical cancer (Fig. 1c). In addition, transcription factor zinc finger protein 667 (Zinc finger protein 667, ZNF667-AS1) is also known as lncRNA MORT. Its expression was significantly low in cervical cancer, and the expression level was negatively correlated with overall survival rate, tumor size and FIGO stage, while high expression of ZNF667-AS1 could reduce the proliferation and clonal ability of cervical cancer cells [16] (Fig. 1d).

### 2.2 LncRNAs affect the invasion and migration of cervical cancer

Invasion is the most critical step in the process of tumor cell metastasis, which includes the degradation of the cell matrix, the activation of tumor cell motility molecular pathways, and the transformation of intercellular links [17]. lncRNAs are essential for promoting cell growth, and their abnormal expression contributes to the growth and survival of tumor cells. Long non-coding ZEB1 antisense 1 (LncRNA ZEB1 Antisense 1, ZEB1-AS1) is upregulated in cervical cancer, and it is associated with the clinical characteristics of cervical cancer invasion and migration. Gan et al. found that the expression of p-p38 could be significantly reduced by interfering with the expression of ZEB1-AS1, indicating that silencing ZEB1-AS1 could effectively inhibit the p38MAPK signaling pathway [18]. Further experiments found that compared with the control group, co-transfection of ZEB1-AS1siRNA and p38MAPK pathway inhibitor SB203580 could inhibit the protein E-cadherin related to epithelial-to-mesenchymal transition (EMT). The expression of Vimentin and N-cadherin which promote EMT transformation, did not change significantly. In addition, after interfering with ZEB1-AS1, the inhibition of EMT transformation in HeLa cells can be reversed by the p38MAPK activator anisomycin, indicating that low expression of ZEB1-AS1 can inhibit the EMT transformation of HeLa cells by blocking the p38MAPK signaling pathway, thereby inhibiting the invasion and migration of HeLa cells (Fig. 1e). In addition, Huang et al. found that Homeobox gene transcript antisense RNA (HOTAIR), a lncRNA highly expressed in cervical cancer, was associated with poor prognosis of cervical cancer [19]. Kim et al. found that after silencing HOTAIR, the expression of VEGF and MMP-9 decreased significantly, the expression of E-cadherin increased, the expression of β-catenin and Vimentin decreased, and the expression of transcription factors Snail and Twist, which promote EMT transformation, decreased, thereby inhibiting the expression of cervical cancer [20]. Both these markers and transcription factors are important players in tumor invasion and migration (Fig. 1f). In addition, after HOTAIR was silenced, the expression of miR206 was up-regulated, while the expression of miR206 downstream target protein megakaryoblastic leukemia 1 (MKL1) was down-regulated, and it could increase the distribution of MKL1 in the cytoplasm, indicating that HOTAIR promotes the expression of MKL1 by inhibiting the expression of miR206 And change the distribution of MKL1 cells to promote cervical cancer invasion and migration. However, MKL1 can bind to the HOTAIR promoter CArG box to activate HOTAIR transcription, and form a positive feedback with HOTAIR to promote HOTAIR expression. In conclusion, MKL1 is an important promoter of HOTAIR in cervical cancer invasion and migration [21] (Fig. 1f).

### 2.3 lncRNAs affect apoptosis and proliferation of cervical cancer

LncRNAs affect the fate of tumor cells by regulating the proliferation and apoptosis of tumor cells. Taurine upregulated gene 1 (TUG1) is an lncRNA upregulated in cervical cancer, which is closely related to the biological characteristics and poor prognosis of cervical cancer cells [22]. Hu et al. found that by experimentally knocking out TUG1, the expression of apoptosis-related mitochondrial pathway protein Bcl-2 was significantly reduced, and the expression of caspase-3 was significantly increased, thereby promoting cervical cancer cell apoptosis [23] (Fig. 1g). In addition, overexpression of cervical cancer highly expressed lncRNA 1 (CCHE1) can promote the proliferation of cervical cancer cells, while knocking out CCHE1 inhibits cell proliferation [24]. RNA pull-down analysis showed that CCHE1 physiologically binds to PCNA mRNA, and the interaction between the two leads to the upregulation of the expression of proliferating cell nuclear antigen (PCNA), a tumor proliferation marker, thereby promoting the proliferation of cervical cancer cells (Fig. 1h). In addition, downregulation of actin filament-associated protein 1-antisense RNA 1 (Actin filament-associated protein 1-antisense RNA1, AFAP1-AS1) and cancer-associated region long non-coding RNA (CARLo-5) can cause cells to undergo S-phase arrest, and the expression of S-phase-related proteins CDK2 and Cyclin A2 will be down-regulated to varying degrees, thereby inhibiting the proliferation of HeLa cells. It was shown that AFAP1-AS1 and CARLo-5 affect cervical cancer proliferation by regulating the cell cycle [25] (Fig. 1i and j).

### 2.4 LncRNAs affect the radiochemotherapy resistance of cervical cancer

More and more studies show that lncRNAs may play a role in chemotherapy and chemotherapy resistance by regulating the cell cycle, apoptosis, and DNA damage repair [[26], [27], [28], [29]]. Eden et al. found that translocation of plasmacytoma variant 1 (PVT1), lncRNAs, significantly highly expressed in cervical cancer, was associated with resistance to the chemotherapy drug cisplatin [30]. At the same time, Shen et al. also found that inhibition of PVT1 expression can significantly increase the expression of E-cadherin in CaSki cells, while the expression of fibronectin and vimentin significantly decreased, thereby increasing the sensitivity of CaSki cells to paclitaxel and high expression PVT1 can induce EMT transformation and make cervical cancer cells resistant to paclitaxel [31]. This suggests that PVT1 promotes paclitaxel resistance in cervical cancer cells, promoting EMT transformation (Fig. 1k). In addition, cancer susceptibility candidate 2 (CASC2) is an lncRNAs with low expression in cervical cancer. Suppression of CASC2 expression can significantly attenuate cisplatin's inhibition of cervical cancer cell proliferation and increase the median lethal dose (IC50), while overexpression can enhance cisplatin's inhibition of cervical cancer cell proliferation and decrease the IC50 value. This suggests that low CASC2 expression contributes to cisplatin resistance in cervical cancer. Mechanistic studies have shown that CASC2 can competitively inhibit miR-21, thereby increasing the miR-21 expression of the downstream target protein PTEN (tumor suppressor), and PTEN can increase the chemosensitivity of cervical cancer cells to cisplatin by regulating the AKT signaling pathway [32] (Fig. 1k). In addition, nuclear-enriched abundant transcript 1 (Nuclear-enriched abundant transcript 1, NEAT1) is a lncRNAs highly expressed in radiation-resistant cervical cancer cells [33]. Suppression of NEAT1 expression attenuated the proliferation of radioresistant cells and reduced the dose of ionizing radiation, while overexpression of NEAT1 did the opposite. This indicates that high expression of NEAT1 is closely related to the resistance of cervical cancer to radiotherapy. Mechanistic studies have shown that after NEAT1 suppression, miR-193b-3p expression is upregulated, cyclin D1 expression is downregulated, resulting in cell cycle arrest in the G0/G1 phase, and caspase-3 and caspase-9 expression is upregulated, inducing apoptosis. This indicated that high expression of NEAT1 contributes to the resistance of cervical cancer cells to radiation therapy through regulation of the cell cycle and apoptosis (Fig. 1m). Thus, in radiotherapy and chemotherapy of cervical cancer, lncRNAs may play a role in resistance to radiotherapy and chemotherapy through a specific molecular regulatory mechanism. Therefore, the regulation of lncRNAs may be a good option for the treatment of patients with cervical cancer, especially those resistant to radiation or chemotherapy.

## 3 Molecular regulation mechanism of lncRNAs in cervical cancer

### 3.1 Interaction of lncRNAs with proteins/mRNAs

LncRNAs can interact with proteins, mRNA or miRNA, and participate in the basic biological functions of living organisms by regulating gene expression, such as gene imprinting, histone modification, mRNA splicing, etc. [34]. Cervical cancer-related lncRNAs can directly bind to proteins or mRNAs to play a regulatory role at the post-transcriptional level. LINC00473 is an lncRNA highly expressed in cervical cancer, and its high expression can promote cervical cancer cell proliferation and inhibit cell apoptosis [35]. Mechanistic studies showed that LINC00473 can directly bind to cell proliferation-related transcription factor interleukin-binding factor 2 (ILF2), which had no effect on ILF2 mRNA levels, but ILF2 protein levels were significantly changed: after silencing LINC00473, the half-life of ILF2 protein was shortened, while overexpression ILF2 protein half-life is prolonged after LINC00473. This indicates that LINC00473 can inhibit ILF2 protein degradation, thereby promoting cervical cancer cell proliferation and inhibiting apoptosis. In addition, the high expression of CCHE1 in cervical cancer can combine with PCNA mRNA to up-regulate the expression of PCNA, thereby promoting the proliferation of cervical cancer cells [24]. These studies suggest that the interaction of lncRNAs with proteins or mRNAs plays a key role in the development of cervical cancer.

### 3.2 Interaction of lncRNAs and miRNAs

The competing endogenous RNA (ceRNA) hypothesis is that lncRNAs, mRNAs, pseudogenes, and circular RNAs in the ncRNAs family competitively bind to miRNAs through their miRNAs response elements (MREs), thereby regulating gene expression. That is to say, lncRNAs can act as ceRNAs to inhibit miRNA expression and activity at the post-transcriptional level [[36], [37], [38]]. Gao et al. reported that the expression of PVT1 was negatively correlated with miR-424, indicating that PVT1 could promote the proliferation, migration and invasion of cervical cancer cells by negatively regulating the expression of miR-424, thereby promoting the development of cervical cancer [39]. In addition, CASC2 can competitively bind to miR-21 and upregulate the expression of PTEN, the downstream target protein of miR-21, thereby promoting the chemosensitivity of cervical cancer cells to cisplatin and inhibiting the development of cervical cancer [32]. In addition, NEAT1 can also act as a ceRNA to inhibit the expression of miR-193b-3p, thereby upregulating the expression of cyclin D1, the downstream target of miR-193b-3p, accelerating the cell cycle and promoting the development of cervical cancer [33]. In summary, lncRNAs can act as a “miRNA sponge” to inhibit the expression of miRNAs, up-regulate or down-regulate the expression of miRNAs downstream targets, thereby promoting or inhibiting the development of tumors.

### 3.3 Single nucleotide polymorphisms (SNPs) of lncRNAs

Genome-wide association studies (GWAS) have revealed a large number of closely related genetic variants associated with diseases and traits, with at least one-third of the identified variants not within protein-coding genes, but instead mapping to non-coding intervals [40]. Verhaegh et al. reported for the first time that the single nucleotide polymorphism of the H19 gene was closely related to the risk of bladder cancer, which opened the prelude to the study of lncRNA single nucleotides and tumors [41]. Multiple studies have shown that somatic mutations such as single nucleotide polymorphisms in tumor suppressor genes or oncogenes play an important role in the genetic susceptibility to cervical cancer [[42], [43], [44]]. Guo et al. conducted a case-control analysis of 510 cervical cancer patients and 713 normal individuals and found that three haplotype SNPs (rs920778, rs1899663 and rs4759314) in HOTAIR were closely related to the risk of cervical cancer [45]. Among them, SNP rs920778 in the HOTAIR enhancer gene was strongly associated with cervical cancer. Compared with the wild-type rs920778 CC genotype, patients carrying the rs920778(CT + TT) mutation genotype had a 2.17-fold increased risk of developing cervical cancer. The HOTAIR rs920778 SNP T variant allele is located in the HOTAIR intron 2 region, which can enhance the activity of the enhancer located in the HOTAIR intron 2 region. And compared with rs920778 CC, HOTAIR mRNA expression was significantly increased in cervical cancer patients carrying rs920778 CT or TT genotype. This indicates that the risk-associated allele T is closely related to the expression of HOTAIR, and SNP rs920778 can promote the expression of HOTAIR, thereby promoting the genetic susceptibility to cervical cancer. At the same time, Jin et al. also reported that HOTAIR rs7958904 affects the genetic susceptibility of cervical cancer by regulating the proliferation of cervical cancer cells [46]. In addition, it has been shown that rs7133268 of the TNF and HNRNPL-related immunoregulatory lncRNA (THRIL) genes can reduce the genetic susceptibility to cervical precancerous lesions [47]. These studies indicate that the single nucleotide polymorphisms of lncRNAs play an important role in the occurrence and development of cervical cancer (Table 1).

**Table 1:** Long non-coding RNAs with oncogenic and oncosuppressive functions in cervical cancer.

LncRNAs	Function	Deregulated pathways in cervical cancer	References
MEG3	Oncosuppressive		[14]
PVT1	Oncogenic	EZH2, Myc, Nop2, p15, p16, H3K27me3, NF-kB	[[48], [49], [50], [51], [52], [53], [54], [55]]
H19	Oncogenic	IGF2, HPV E6 oncoprotein	[56]
FAM83H-AS1	Oncogenic	HPV E6, E6-p300	[57]
MALAT1	Oncogenic	RBG2, E-cadherin, β-catenin, vimentin, ZO-1, caspase-3, caspase-8, Bax, Bcl-2, and BclxL	[58]
PAX8 AS1	Oncosuppressive	PAX8, NOTCH1 (pancreatic carcinoma)	[59]
CCAT2	Oncogenic	MYC, wnt in colon cancer	[60]
C5orf66-AS1	Oncogenic	RING1	[61]
SPRY4-IT1	Oncogenic	ZEB1, EMT, E-cadherin, vimentin	[62]
CCAT1	Oncogenic	MMP14	[63]
GAS5	Oncosuppressive	IER3	[64]
NOC2L-4.1	Oncogenic	YAP1	[65]
CCHE1	Oncogenic	PCNA, ERK/MAPK	[24,66]
HOTAIR	Oncogenic	BCL2, PRC2, LSD1, VEGF, mmP-9, mTOR, Notch, Wnt, STAT3, wnt/β-catenin, PI3K/AKT, HPV E7 oncoprotein	[19,20,[67], [68], [69], [70], [71], [72], [73], [74], [75]]
EBIC	Oncogenic	EZH2, Wnt/β-catenin, E-cadherin	[76,77]
RSU1P2	Oncogenic	IGF1R, N-myc	[78]
LINC00675	Oncogenic	Wnt/β-catenin, Bax and GSK-3β Bcl-2	[79]

## 4 Potential clinical application of lncrnas in cervical cancer

Metastasis and recurrence are the biggest obstacles in the clinical treatment of cervical cancer. Therefore, finding effective tumor markers is of great value in improving the prognosis of cervical cancer. LncRNAs can be used in the diagnosis and prognosis of cervical cancer. For example, receiver operating characteristic curve (ROC) analysis showed that the expression of SPRY4 intronic transcript 1 (SPRY4 intronic transcript 1, SPRY4-IT1) is a good candidate for distinguishing cervical cancer tissue from normal tissue (sensitivity: 78.3%, specificity: 63.6%), the area under the ROC curve (Area under ROC curve, AUC) was 0.741 (95%CI: 0.632–0.849, P < 0.001), indicating that the diagnostic accuracy of SPRY4 for cervical cancer was higher than that of high [80]. In addition, HOTAIR can also be used to distinguish cervical cancer tissue from normal tissue (sensitivity: 60.6%, heterosexuality: 87.2%) and lymph node metastasis (sensitivity: 85.1%, specificity: 64.9%), and the multivariate Cox regression model showed FIGO stage (P < 0.000 1, HR = 1.994, 95%CI: 1.359–2.927), lymph node metastasis (P = 0.005, HR = 2.636, 95%CI: 1.348–5.156) and HOTAIR expression level (P = 0.012, HR = 2.863, 95% CI: 1.263–6.490), indicating that HOTAIR has high diagnostic accuracy for cervical cancer and can be used as an independent predictor for the prognosis of cervical cancer [19]. In summary, SPRY4 and HOTAIR among lncRNAs are promising markers of cervical cancer, which can be used as diagnostic indicators and good markers for prognosis of cervical cancer (Table 2).

**Table 2:** Potential of using lncRNAs in cervical cancer therapy.

LncRNA	Involved cases (no. and sample)	Detection material	Clinical marker type and Clinical signiﬁcance	References
SOX21-AS1	160 patients (tumor tissue vs. adjacent normal tissue)	Tissue	Prognostic High SOX21-AS1 has a shorter OS and is positively correlated with FIGO stage, lymph node metastasis, and depth of cervical invasion	[81]
MEG3	Plasma (36 CIN I, 48 CIN II, 76 CIN III,168 cervical cancer patients, 328 healthy controls) Tissue (168 cervical cancer tissue vs. adjacent normal tissue)	Plasma	Diagnostic and Prognostic Higher MEG3 methylation level is associated with poor RFS and OS and positively correlated HR-HPV infection, lymph node metastasis. The sensitivity and speciﬁcity are 81.3% and 63.5% for prediction of lymph node metastasis, 63.1% and 84.2% for the diagnosis of HPV infection.	[82]
GHET1	94 patients with cervical cancer vs. 47 normal	Tissue	Prognostic High GHET1 is associated with poor OS and correlated with clinical stage, distant metastasis, and poor	[83]
SNHG14	30 patients (tumor tissue vs. adjacent normal tissue)	Tissue	Prognostic High SNHG14 has a shorter OS and is positively correlated with tumor size, FIGO stage, and lymph node metastasis	[84]
LINC00511	92 cervical cancer tissues vs. 40 adjacent normal tissues	Tissue	Prognostic High LINC00511 is associated with poor OS and positively correlated with clinical stage, tumor size, lymph node metastasis, histological type of adenocarcinoma, and distant metastasis	[85]
PVT1	156 patients with SCCs (tumor tissue vs. adjacent normal tissue)	Serum and tissues Prognostic	Diagnostic and Prognostic High serum PVT1 has a shorter OS and is positively correlated with tumor size	[86]
AFAP1-AS1	TCGA, SRA, GEO and UCSC XENA database	Tissue	Prognostic High AFAP1-AS1 is positively associated with the TNM stage, high expression and hypomethylation of AFAP1-AS1 is associated with poor OS	[87]
lncRNA-ATB	187 patients (tumor tissue vs. adjacent normal tissue)	Tissue	Prognostic High lncRNA-ATB has a shorter OS and PFS and is positively correlated with SCC antigen level, tumor size, lymph node metastasis, and FIGO stage	[88]
GIHCG	Plasma (80 patients with cervical cancer vs. 80 normal subjects) Tissue (58 cervical cancer tissue vs. adjacent normal tissue)	Serum and tissue	Diagnostic The sensitivity and speciﬁcity is 88.75% and 87.5% for cervical cancer diagnosis	[89]
AC126474	TCGA database (305 patients with cervical cancer vs. 305 normal subjects)	Tissue	Prognostic Low AC126474 and C5orf66-AS1 are associated with poor OS	[90]

## 5 Conclusion

LncRNAs pass through the entire biological process, are involved in the regulation of cancer cell proliferation, anti-apoptosis, migration, and resistance to radiation and chemotherapy through various molecular regulatory mechanisms, and play a key role in the development of various forms of cancer, including cervical cancer [[91], [92], [93], [94]]. Therefore, these lncRNAs are ideal molecular markers for cervical cancer and are expected to be effective targets for the treatment of cervical cancer. Although some progress has been made in the study of lncRNA in the pathological mechanism of cervical cancer development, the occurrence and development of cervical cancer is determined not by any one factor, but by a combination of other factors that affect the incidence of cervical cancer. For example: studies of lncRNAs and epigenetics, including DNA methylation, histone modification, gene imprinting, chromosome remodeling, etc.; study of regulatory networks of lncRNAs and microRNAs, study of cell signal transduction and lncRNAs pathways, etc. The focus of future research should be based on existing research results, further deepen the study of the lncRNA mechanism that regulates the occurrence and development of cervical cancer, and fully solve the mystery. LncRNAs will have broad prospects for use as effective markers for the clinical diagnosis and treatment of cervical cancer.

## Funding

This work was supported by the Bashkir State Medical University Strategic Academic Leadership Program (PRIORITY-2030).

## Author contributions

Sema Begliarzade, Albert Sufianov and Rasim Tamrazov conceptualized and designed the study. All authors have participated in the acquisition, analysis and interpretation of the data. Aferin Beilerli, Valentin Kudriashov has drafted the manuscript. Tatiana Ilyasova and Yanchao Liang contributed to the critical revisions of the manuscript. Ozal Beylerli supervised the research. All authors agreed on the journal to which the article would be submitted, gave the final approval for the version to be published, and agreed to be accountable for all aspects of the work.

## Declaration of competing interest

The authors declare they have no conflict of interest.
